# Pharmacokinetic Interactions between Tafenoquine and Dihydroartemisinin-Piperaquine or Artemether-Lumefantrine in Healthy Adult Subjects

**DOI:** 10.1128/AAC.01588-16

**Published:** 2016-11-21

**Authors:** Justin A. Green, Khadeeja Mohamed, Navin Goyal, Samia Bouhired, Azra Hussaini, Siôn W. Jones, Gavin C. K. W. Koh, Ivan Kostov, Maxine Taylor, Allen Wolstenholm, Stephan Duparc

**Affiliations:** a GlaxoSmithKline Research and Development, Stockley Park West, Uxbridge, Middlesex, United Kingdom; b GlaxoSmithKline, Upper Merion, King of Prussia, Pennsylvania, USA; c Parexel, Harbor Hospital, Baltimore, Baltimore, Maryland, USA; d GlaxoSmithKline Research and Development, Ware, Hertfordshire, United Kingdom; e GlaxoSmithKline, Upper Providence, Collegeville, Pennsylvania, USA; f Medicines for Malaria Venture, Geneva, Switzerland

## Abstract

Tafenoquine is in development as a single-dose treatment for relapse prevention in individuals with Plasmodium vivax malaria. Tafenoquine must be coadministered with a blood schizonticide, either chloroquine or artemisinin-based combination therapy (ACT). This open-label, randomized, parallel-group study evaluated potential drug interactions between tafenoquine and two ACTs: dihydroartemisinin-piperaquine and artemether-lumefantrine. Healthy volunteers of either sex aged 18 to 65 years without glucose-6-phosphate dehydrogenase deficiency were randomized into five cohorts (*n* = 24 per cohort) to receive tafenoquine on day 1 (300 mg) plus once-daily dihydroartemisinin-piperaquine on days 1, 2, and 3 (120 mg/960 mg for 36 to <75 kg of body weight and 160 mg/1,280 mg for ≥75 to 100 kg of body weight), or plus artemether-lumefantrine (80 mg/480 mg) in two doses 8 h apart on day 1 and then twice daily on days 2 and 3, or each drug alone. The pharmacokinetic parameters of tafenoquine, piperaquine, lumefantrine, artemether, and dihydroartemisinin were determined by using noncompartmental methods. Point estimates and 90% confidence intervals were calculated for area under the concentration-time curve (AUC) and maximum observed plasma concentration (*C*_max_) comparisons of tafenoquine plus ACT versus tafenoquine or ACT. All subjects receiving dihydroartemisinin-piperaquine experienced QTc prolongation (a known risk with this drug), but tafenoquine coadministration had no clinically relevant additional effect. Tafenoquine coadministration had no clinically relevant effects on dihydroartemisinin, piperaquine, artemether, or lumefantrine pharmacokinetics. Dihydroartemisinin-piperaquine coadministration increased the tafenoquine *C*_max_ by 38% (90% confidence interval, 25 to 52%), the AUC from time zero to infinity (AUC_0–∞_) by 12% (1 to 26%), and the half-life (*t*_1/2_) by 29% (19 to 40%), with no effect on the AUC from time zero to the time of the last nonzero concentration (AUC_0–last_). Artemether-lumefantrine coadministration had no effect on tafenoquine pharmacokinetics. Tafenoquine can be coadministered with dihydroartemisinin-piperaquine or artemether-lumefantrine without dose adjustment for any of these compounds. (This study has been registered at ClinicalTrials.gov under registration no. NCT02184637.)

## INTRODUCTION

The protozoan parasite Plasmodium vivax caused an estimated 13.8 million malaria cases globally in 2015, accounting for approximately half of the total number of malaria cases outside Africa (1). Plasmodium vivax has a parasite life cycle with a dormant liver stage, the hypnozoite, which is undetectable by using currently available diagnostic methods. The hypnozoite stage allows P. vivax to survive in the host following antimalarial therapy, eventually reemerging to cause repeated clinical malaria episodes (relapses), weeks, months, or even years later. Not only do relapses represent a significant burden of morbidity, they also maintain P. vivax transmission, allowing the parasite to evade conventional malaria control measures.

The only treatment available for the prevention of P. vivax relapse is the 8-aminoquinoline antihypnozoite agent primaquine. Primaquine is given in combination with the schizonticide chloroquine, which is required to clear acute blood-stage infection and to potentiate the antihypnozoite effect of primaquine. Chloroquine is given over 3 days, but primaquine dosing recommendations require 0.25 to 0.5 mg/kg of body weight daily for 14 days (2), and treatment effectiveness is compromised by the lack of adherence to this regimen (3).

Tafenoquine is a long-acting synthetic analogue of primaquine currently in phase III clinical development for the prevention of P. vivax relapse. In phase II studies, 89% (95% confidence interval [CI], 77 to 95%) of patients receiving a single 300-mg dose of tafenoquine in combination with standard 3-day chloroquine treatment were relapse-free at 6 months, compared with 77% (63 to 87%) of those receiving 15 mg/day primaquine for 14 days plus chloroquine and 38% (23 to 52%) of those who received chloroquine alone (4).

The most significant risk for both primaquine and tafenoquine treatment is hemolysis in individuals who are glucose-6-phosphate dehydrogenase (G6PD) deficient (5, 6). G6PD deficiency is an X-linked inherited condition widespread in areas where malaria is endemic, with an estimated overall prevalence of 8%, although this may be much higher in some populations (up to 33%) (7). Therefore, the most recent malaria treatment guidelines recommend pretreatment G6PD testing to exclude patients at the highest risk for hemolysis (2). Additionally, 8-aminoquinolines are known to increase methemoglobin levels when given at high doses, although clinical signs of methemoglobinemia are rare (8, 9).

Plasmodium vivax malaria is treated with chloroquine in areas where this medicine remains effective. However, high-grade chloroquine resistance has been confirmed in P. vivax in Papua New Guinea and Indonesia (10). Evidence of declining chloroquine efficacy in other areas where P. vivax is endemic is also accumulating, although surveillance is inadequate (10). In areas where chloroquine-resistant P. vivax has been identified, artemisinin-based combination therapies (ACTs) have been adopted as first-line therapy for all malaria species. Thus, for the prevention of P. vivax relapse, tafenoquine will need to be given in combination with either chloroquine or ACTs, depending on the resistance situation and local guidelines.

In a previous drug interaction study, no clinically significant safety or pharmacokinetic (PK) interactions were observed for tafenoquine coadministered with chloroquine in healthy subjects (11). The present study aimed to characterize any pharmacokinetic drug interactions between tafenoquine and two widely used first-line ACTs, dihydroartemisinin-piperaquine (DHA-PQP) and artemether-lumefantrine, in healthy subjects. These data will support clinical trials of tafenoquine plus ACTs in regions where chloroquine-resistant P. vivax has been identified or in countries where the first-line treatment for P. vivax is an ACT.

## MATERIALS AND METHODS

### Study design.

**FIG 1:**
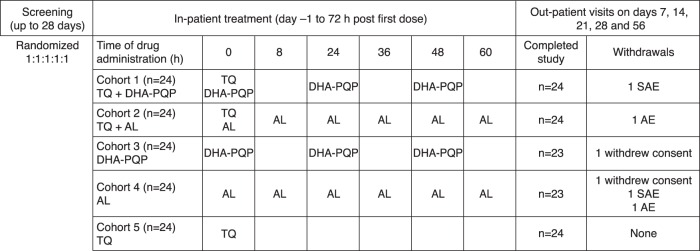
Study design and drug dosing. TQ, tafenoquine; DHA-PQP, dihydroartemisinin-piperaquine; AL, artemether-lumefantrine; AE adverse event; SAE, serious adverse event.

This was an open-label, randomized, five-cohort, parallel-group study conducted with healthy volunteers at a single study center (Parexel Early Phase Unit, Baltimore, MD, USA) (Fig. 1). A parallel design was chosen because of the long half-lives of tafenoquine (15 to 19 days), piperaquine (22 days), and lumefantrine (5 days). The study was approved by the Aspire Institutional Review Board and conducted according to ICH good clinical practice guidelines, the Declaration of Helsinki (2008), and all applicable regulatory requirements. Written informed consent was obtained from all subjects. This study has been registered at ClinicalTrials.gov under registration no. NCT02184637.

### Study subjects.

Eligible subjects were healthy volunteers of either sex, aged between 18 and 65 years, with a body mass index of 18.5 to 31.0 km/m^2^ and a body weight of between ≥36 and ≤100 kg. Additional inclusion criteria were alanine aminotransferase, alkaline phosphatase, and bilirubin values ≤1.5× the upper limit of normal (ULN), a Fridericia-corrected QT interval (QTcF) of <450 ms based on triplicate electrocardiograms (ECGs), and no additional cardiac risk factors for Torsades de Pointes. Female subjects had to be nonlactating and either without childbearing potential or with agreement to use approved methods of contraception and undergo a pregnancy test at screening and at enrollment, before drug treatment.

Exclusion criteria were G6PD deficiency (determined as G6PD activity of <70% of a locally defined median, i.e., 10.7 IU/g hemoglobin); history of thalassemia or methemoglobinemia; blood pressure outside the range of 80 to 140 mm Hg for systolic or 45 to 90 mm Hg for diastolic blood pressure; hemoglobin levels outside the normal range; potassium levels of <4.0 mmol/liter; magnesium levels of <1.6 mg/dl (amended during the study from <1.8 mg/dl); positive hepatitis B surface antigen or hepatitis C antibody result within 3 months of screening; positive HIV antibody test; history of liver disease, known hepatic or biliary abnormalities, or regular alcohol consumption; cotinine levels indicative of smoking or history of regular tobacco use; history of heparin sensitivity or heparin-induced thrombocytopenia; history of sensitivity to study drugs; participation in a clinical trial or receipt of an investigational drug within 30 days or 5 half-lives of the start of the study, exposure to more than four new chemical entities within the previous 12 months; consumption of citrus fruits for 7 days prior to the start of the study; use of other prescription or nonprescription drugs (except oral contraceptives); and use of herbal and dietary supplements within 7 days (14 days if the product is a potential enzyme inducer) or 5 half-lives of the start of the study.

### Study drug administration.

Study treatments were tafenoquine (150 mg per tablet) (GlaxoSmithKline), dihydroartemisinin-piperaquine tetraphosphate (40 mg/320 mg per tablet) (Eurartesim; Sigma-Tau Industrie Farmaceutiche Riunite SPA), and artemether-lumefantrine (20 mg/120 mg per tablet) (Coartem; Novartis Pharmaceuticals). Subjects were randomized into five cohorts (*n* = 24 per cohort) to receive tafenoquine on day 1 (300 mg) plus once-daily dihydroartemisinin-piperaquine on days 1, 2, and 3 (120 mg/960 mg for subjects with a body weight of 36 to <75 kg and 160 mg/1,280 mg for subjects with a body weight of ≥75 to 100 kg), or plus artemether-lumefantrine (80 mg/480 mg) with the second dose administered 8 h after the first dose on day 1 and then twice daily on days 2 and 3, or each drug given alone (Fig. 1). No dose adjustments were allowed.

### Study procedures.

Subjects were screened for eligibility from 28 days to 2 days before enrollment and observed as inpatients from day 1 until 72 h post-first dose (day 3). Outpatient visits were scheduled for days 7, 14, 21, 28, and 56. On days 1, 2, and 3, subjects were given a standard breakfast at a time that allowed them to finish eating just prior to study drug administration. Note that taking dihydroartemisinin-piperaquine with food does not comply with dihydroartemisinin-piperaquine tetraphosphate dosing recommendations (i.e., in order to reduce the potential for QT interval prolongation, each dose should be taken no less than 3 h after the last food intake, and no food should be taken within 3 h after each dose). However, based on previous clinical trials, tafenoquine has been administered with food to avoid any gastrointestinal tolerability issues. Thus, a low-fat meal was chosen for this study to provide a dietary recommendation that would be feasible for use in the clinical-trial setting and to anticipate real-world drug administration where medications may be taken with food despite recommendations. Lunch and dinner were provided only after any scheduled pharmacokinetic blood samples were obtained. All meal times during the 72-h-post-first-dose inpatient phase were consistent across cohorts. Subjects had to abstain from ingesting caffeine- or xanthine-containing products or alcohol for 24 h prior to the start of dosing until 72 h post-first dose and citrus fruits for 7 days before the first dose until 72 h post-first dose. Tobacco use was not allowed from screening until the final follow-up visit (day 56). Subjects were to abstain from strenuous exercise for 48 h prior to each blood sample collection. Dried blood spots were collected for G6PD genotyping.

### Adverse events and laboratory assessments.

Physical examination was performed, medical history was taken at screening, and physical examination was repeated at days 4 and 56. Adverse events and serious adverse events were noted during screening, at the start of drug treatment on day 1, and at all subsequent visits and coded by using the MedDRA dictionary version 12.0 for all entries plus version 17.1 for four individual entries. Serious adverse events were those resulting in death, that were life-threatening, that resulted in disability/incapacity, hospitalization or prolongation of hospitalization, congenital abnormality, or liver injury plus impaired liver function, or that were considered serious by the investigator. In addition, any hemoglobin decline of ≥3.0 g/dl or 30% from the baseline (mean of predose and day −1 results) associated with clinical evidence of hemolysis and no other explanation was recorded as a serious adverse event, and the subject was monitored until resolution.

Hematology, clinical chemistry, and urinalysis testing were performed at screening and at predosing on day 1; on days 2, 3, 4, 7, 14, and 28; and where clinically indicated. Hematology testing included a full blood count, reticulocyte count, hemoglobin level determination, hematocrit determination, and percent methemoglobin determination. Clinical chemistry included liver function tests (the ULN values were 41 IU/liter for alanine aminotransferase, 34 IU/liter for aspartate aminotransferase, and 308 IU/liter for males and 192 IU/liter for females for creatine kinase) and determination of levels of total and direct bilirubin, blood urea nitrogen/creatinine, blood glucose, electrolytes, CO_2_, uric acid, albumin, and total protein. Study treatment was stopped if the alanine aminotransferase level was ≥3× the ULN.

### Pharmacodynamic evaluation.

Triplicate 12-lead ECGs and vital signs were taken at screening; at predosing on day 1; and then singly at 12, 24, 36, 48, 60, and 72 h post-first dose plus at 52 h post-first dose for cohorts 1 and 3 and at days 7, 14, 28, and 56. Continuous cardiac telemetry was performed for cohorts 1 to 4 for at least 15 min prior to the first dose until 72 h post-first dose. If a subject had a QTcF of >480 ms at any time, the subject was to be monitored by telemetry until resolution. If an ECG demonstrated a prolonged QT interval, two more ECGs were obtained over a brief period, and study withdrawal was triggered by a QTcF of >500 ms or a change compared to the baseline QTcF of >60 ms with an absolute value of >480 ms, based on the average for triplicate ECGs. The change in the QTcF from the baseline over time was analyzed by mixed-effect models, with treatment, time, and the time-by-treatment interaction as fixed effects and subject as a random effect. The baseline QTcF was fitted as a covariate, and an unstructured covariance structure was used for repeated measures. Models were fitted separately to assess the interaction of tafenoquine with each ACT and point estimates of treatment differences, and corresponding two-sided 90% confidence intervals were determined for each ACT. The maximum change in the QTcF from the baseline was evaluated for each cohort and for cohorts receiving piperaquine; the change from the baseline at 52 h post-first dose was also analyzed. The baseline measurement was the average of triplicate measurements taken predose on day 1. The maximum change in QTcF from the baseline was analyzed by using an analysis-of-covariance model using the baseline QTcF as a covariate.

### Pharmacokinetic assessments.

Blood samples (2 ml) for pharmacokinetic analysis were collected into tubes containing K3EDTA anticoagulant at time points relevant for the concentration-time curves of the experimental drugs (see Table SA1 in the supplemental material). Each sample was centrifuged at 1,500 × *g* for 10 min in a refrigerated centrifuge (4°C) to produce plasma and stored at −80°C (−20°C for tafenoquine).

Plasma samples were analyzed for concentrations of tafenoquine, piperaquine, dihydroartemisinin, artemether, or lumefantrine by Aptuit (Verona) SRL, Verona, Italy. Validated analytical methods were used based on protein precipitation or liquid-liquid extraction, followed by-high pressure liquid chromatography (HPLC) or ultra-high-pressure liquid chromatography (UHPLC) and tandem mass spectrometry with a Turbolonspray interface with positive-ion multiple-reaction monitoring. The lower limit of quantification (LLQ) and the higher limit of quantification (HLQ) for tafenoquine, dihydroartemisinin, artemether, piperaquine, and lumefantrine were 2 and 3,000 ng/ml, 1 and 2,000 ng/ml, 5 and 2,000 ng/ml, 2 and 2,000 ng/ml, and 4 and 4,000 ng/ml, respectively, using 25-μl, 125-μl, 125-μl, 100-μl, and 50-μl aliquots of K3EDTA-treated plasma, respectively.

For each analytical method, quality control (QC) samples that contained tafenoquine, piperaquine, dihydroartemisinin, artemether, or lumefantrine at concentrations spanning the calibration range of the method and that were stored with study samples were analyzed with each batch of samples against separately prepared calibration standards. The analysis was acceptable when no more than one-third of the QC results deviated from the nominal concentration by >15%, and at least 50% of the results from each QC concentration had to be within 15% of nominal value. Concentrations in the plasma samples were then calculated from the calibration plots. A weighted 1/*x*^2^ linear regression was applied in each case over the range of the lower to higher limits of quantification detailed above.

### Pharmacokinetic analysis.

Plasma concentration-time data were analyzed by noncompartmental methods using linear up-log down interpolation with Phoenix WinNonlin v.6.2.1 (Certara, Princeton, NJ), based on observed data. The following pharmacokinetic parameters were determined: maximum observed plasma concentration (*C*_max_), time to *C*_max_ (*T*_max_), area under the plasma concentration-time curve (AUC) (total exposure during the dosing interval [AUC_0–τ_], predicted AUC from time zero to infinity [AUC_0–∞_], and AUC from time zero to the time of the last nonzero concentration [AUC_0–last_]), and apparent terminal-phase half-life (*t*_1/2_). The AUCs were calculated over the following time windows: 28 days (672 h) for artemether, dihydroartemisinin (metabolite), and lumefantrine and 56 days (1,344 h) for dihydroartemisinin, piperaquine, and tafenoquine.

The primary outcome measure was the ratio of the geometric mean changes in *C*_max_ and AUC (AUC_0–τ_, AUC_0–∞_, or AUC_0–last_) for treatment comparisons of tafenoquine plus ACT (dihydroartemisinin-piperaquine or artemether-lumefantrine) versus tafenoquine and of tafenoquine plus ACT versus ACT, determined as follows. Following log_*e*_ transformation, the *C*_max_ and AUC were determined separately for tafenoquine, piperaquine, artemether, dihydroartemisinin, and lumefantrine. Analysis-of-variance models with a single term for treatment were fitted separately for each analyte. Point estimates and their associated 90% CIs were constructed for differences between tafenoquine plus ACT (test) and either tafenoquine or ACT (reference). The point estimates and their associated 90% CIs were then exponentially back-transformed to provide point estimates and 90% CIs for the ratios of geometric means for the treatment comparisons. No formal prespecified hypothesis tests were undertaken. A 90% CI for the geometric mean ratio of the *C*_max_ and AUC for the comparisons of interest that excluded a value of 1.00 was interpreted as representing a difference between the test and the reference. Bioequivalence was inferred if the 90% CI for the geometric mean ratio of the *C*_max_ or AUC was within the range of 0.8 to 1.25 (12).

For secondary outcomes, an analysis similar to that described above was performed to determine changes in *t*_1/2_ values. The *T*_max_ was analyzed by using the Hodges-Lehmann nonparametric method (13) to compute point estimates and associated 90% confidence intervals for the median differences for treatment comparisons of tafenoquine plus ACT (dihydroartemisinin-piperaquine or artemether-lumefantrine) versus tafenoquine and of tafenoquine plus ACT versus ACT.

Distributional assumptions underlying the statistical analyses were assessed by visual inspection of residual plots. Normality was examined by normal probability plots, and homogeneity of variance was assessed by plotting the residuals against the predicted values for the model.

### Sample size.

Using the estimation-based approach, and assuming between-subject coefficients of variation as high as 70% for the AUC and *C*_max_ for some of the artemisinin components, it was estimated that a sample size of 22 evaluable subjects per cohort would provide a precision of 27% for the treatment comparisons of AUC and *C*_max_. The between-subject coefficient of variation for tafenoquine is ∼40%. For an estimated point estimate of a geometric mean ratio for the test/reference of 1.0, the 90% CIs would be 0.73 to 1.38. These calculations are based on the natural log scale and a two-tailed procedure with a type I error rate of 10%. No adjustments for preplanned multiple comparisons were made. Allowing for subject withdrawals, target enrollment was 24 subjects per cohort (120 subjects in total).

## RESULTS

### Subjects.

Of the 120 subjects enrolled, 118 completed the study, with 2 subjects withdrawing consent (Fig. 1). Subject baseline data were generally similar across the five cohorts (Table 1). All subjects were included in the safety population. All subjects, including those with partial pharmacokinetic data, were included in the pharmacokinetic analysis, although primary analyses were conducted on those who also completed the dosing regimen.

**TABLE 1:** Demographic characteristics and predose laboratory summary statistics for study subjects in each treatment group^*a*^

Characteristic or statistic	Value for treatment group				
	TQ + DHA-PQP (*n* = 24)	TQ + AL (*n* = 24)	DHA-PQP (*n* = 24)	AL (*n* = 24)	TQ (*n* = 24)
Mean age (yr) (SD)	40.0 (11.8)	39.5 (10.4)	38.3 (11.2)	35.8 (13.4)	35.8 (10.7)
Mean wt (kg) (SD)	78.6 (12.0)	78.3 (9.5)	76.8 (10.4)	76.0 (12.1)	76.3 (11.5)
Mean ht (cm) (SD)	174.5 (9.8)	173.9 (8.5)	176.5 (8.7)	172.6 (10.0)	173.6 (9.2)
No. of male subjects/no. of female subjects	19/5	19/5	21/3	17/7	18/6
No. (%) of subjects of race					
Caucasian	5 (21)	8 (33)	7 (29)	5 (21)	2 (8)
Black or African American	18 (75)	13 (54)	16 (67)	19 (79)	21 (88)
Other	1 (4)	3 (12)	1 (4)	0	1 (4)
Mean G6DP enzyme activity (U/g Hb) (SD)	10.6 (1.6)	10.4 (1.5)	10.0 (1.1)	9.9 (1.1)	10.2 (1.0)
Mean hemoglobin level (g/dl) (SD)	13.8 (1.1)	13.8 (1.4)	14.1 (1.0)	13.8 (1.5)	13.8 (1.3)
Mean methemoglobin level (%) (SD)	0.99 (0.33)	1.00 (0.28)	0.95 (0.30)	0.98 (0.37)	1.13 (0.32)
Mean creatinine level (μmol/dl) (SD)	8.2 (1.7)	8.5 (1.8)	8.5 (1.4)	8.1 (1.5)	8.4 (1.5)
Mean albumin level (g/dl) (SD)	3.8 (0.3)	3.8 (0.3)	3.8 (0.2)	3.8 (0.3)	3.8 (0.3)
Mean ALT level (U/liter) (SD)	25.8 (8.9)	25.3 (11.8)	23.6 (9.4)	22.8 (8.3)	21.5 (7.6)
Mean AST level (U/liter)	20.1 (7.7)	17.3 (4.7)	17.1 (3.5)	23.1 (28.0)	18.3 (4.3)

a TQ, tafenoquine; DHA-PQP, dihydroartemisinin-piperaquine; AL, artemether-lumefantrine; ALT, alanine aminotransferase; AST, aspartate aminotransferase; Hb, hemoglobin.

### Adverse events.

There were no clinically relevant changes from the baseline for vital signs or physical examination. There was no apparent effect of the addition of tafenoquine on the number of patients experiencing adverse events of any cause compared with ACTs alone: 58.3% (14/24) for dihydroartemisinin-piperaquine versus 37.5% (9/24) for tafenoquine plus dihydroartemisinin-piperaquine and 54.2% (13/24) for artemether-lumefantrine versus 33.3% (8/24) for tafenoquine plus artemether-lumefantrine, compared with 12.5% (3/24) for tafenoquine alone. Headache was the most common adverse event with tafenoquine-containing regimens (8/72), and the severity was mild (*n* = 6) or moderate (*n* = 2). Except for a possible increase in the incidence of headache with tafenoquine plus artemether-lumefantrine (4/24) versus artemether-lumefantrine (1/24), there was no discernible effect of the addition of tafenoquine on the nature of the adverse events experienced compared with ACTs alone (Table 2).

**TABLE 2:** Summary of adverse events due to any cause occurring in >1 subject^*a*^

Adverse event	No. of subjects with adverse event in treatment group				
	TQ + DHA-PQP (*n* = 24)	TQ + AL (*n* = 24)	DHA-PQP (*n* = 24)	AL (*n* = 24)	TQ (*n* = 24)
Fatigue	1	1	0	1	0
Influenza-like illness	1	1	1	0	0
Medical device site reaction	1	0	2	0	0
Asthenia	0	1	1	0	0
Feeling hot	1	0	1	0	0
Pyrexia	1	1	0	0	0
Headache	3	4	3	1	1
Dizziness	0	2	1	0	0
Nausea	2	1	2	0	0
Diarrhea	1	1	2	0	0
Abdominal pain	1	1	0	0	0
Contact dermatitis	2	0	1	1	0
Ventricular tachycardia	0	0	1	1	0
Upper RTI	0	0	0	1	1
Throat irritation	0	0	1	1	0
Decreased appetite	0	1	1	1	0

a TQ, tafenoquine; DHA-PQP, dihydroartemisinin-piperaquine; AL, artemether-lumefantrine; RTI, respiratory tract infection.

Drug-related adverse events occurred in 1/24 (4.2%) subjects receiving tafenoquine alone (mild severity). Drug-related adverse events occurred in 33.3% (8/24) of subjects receiving dihydroartemisinin-piperaquine (all mild except for one case of moderate headache) and in 16.7% (4/24) of subjects receiving tafenoquine plus dihydroartemisinin-piperaquine (all mild). Drug-related headache was more common with tafenoquine plus artemether-lumefantrine (16.7%; 4/24) than with artemether-lumefantrine (4.2%; 1/24), and this explained the increased rate of drug-related adverse events in the cohort that received tafenoquine plus artemether-lumefantrine (33.3%; 8/24) versus the artemether-lumefantrine cohort (25.0%; 6/24). In the cohort treated with tafenoquine plus artemether-lumefantrine, three subjects had drug-related adverse events of moderate severity (nausea/abdominal pain, headache/joint stiffness, and headache/dysuria), and the remainder were of mild severity. In the artemether-lumefantrine cohort, two subjects had drug-related adverse events of moderate severity (increased levels of hepatic enzymes and rhabdomyolysis), and the remainder were of mild severity.

There were no deaths in the study. In total, there were four treatment withdrawals caused by adverse events. Two subjects experienced serious adverse events and were withdrawn from the study. There was one serious adverse event of cardiac arrest in a 36-year-old male subject in the dihydroartemisinin-piperaquine group, which was considered drug related. Two days after receiving the first dose of dihydroartemisinin-piperaquine, during a meal, the subject developed severe (grade 3) asystole. Upon cardiac telemetry, the subject had irregular bradycardia followed by asystole for 43 s, and cardiopulmonary resuscitation was initiated. Postarrest, vital signs included a blood pressure of 118/62 mm Hg and a heart rate of 66 beats per min. The ECG showed normal sinus rhythm and was essentially a normal ECG. Review of the baseline ECG revealed bradycardia of 50 beats per min with no other cardiac abnormalities. Retrospectively, the subject stated that he had diarrhea, but there was no other signs of infection. This was assessed by a local cardiologist at the time of the event as being most likely caused by high vagal tone, nausea, and diarrhea or a combination of a reaction to the study drug and his resting bradycardia. There was one serious adverse event in the artemether-lumefantrine group of moderate ventricular tachycardia in a subject with undisclosed preexisting palpitations upon exertion, which was not considered drug related. The remaining two treatment withdrawals were one case of dry eyes, classified as mild, in the artemether-lumefantrine group (not drug related) and one case of mild vomiting/fever/anorexia/dysuria and moderate headache in the group that received tafenoquine plus artemether-lumefantrine (drug related). There was an additional serious adverse event that did not lead to study withdrawal: a subject with undisclosed smoking and asthma who received dihydroartemisinin-piperaquine had pneumonia that was not thought to be treatment related. All of the above-described five subjects completed the study and recovered fully. There were no serious adverse events in any subject receiving tafenoquine.

### Laboratory assessments.

There was no apparent effect of tafenoquine coadministration with ACTs on any indicators of liver function. Two subjects who received artemether-lumefantrine alone had increased liver transaminase levels: a 56-year-old male had elevated alanine aminotransferase levels (135 IU/liter; grade 2) and aspartate aminotransferase levels (70 IU/liter; grade 1) on day 7, both of which returned to normal by day 11, and normal bilirubin levels; and a 23-year-old male had elevated alanine aminotransferase levels, ranging between 124 and 318 IU/liter on days 7 to 14 (grade 3); elevated aspartate aminotransferase levels (490 to 892 IU/liter; grade 4) on days 7 to 9, with values returning to normal by day 21; and normal bilirubin levels. Additionally, in this subject, the creatine kinase level reached a peak of 50,115 IU/liter on day 8 (grade 4) but had decreased to 430 IU/liter by day 21. The subject was diagnosed with asymptomatic exercise-induced rhabdomyolysis, and this subject's laboratory values returned to normal, with no clinical sequelae. For creatinine and electrolyte levels, there were no changes outside the reference range for these parameters in any cohort and no additional effect of coadministration of tafenoquine and ACTs.

**FIG 2:**
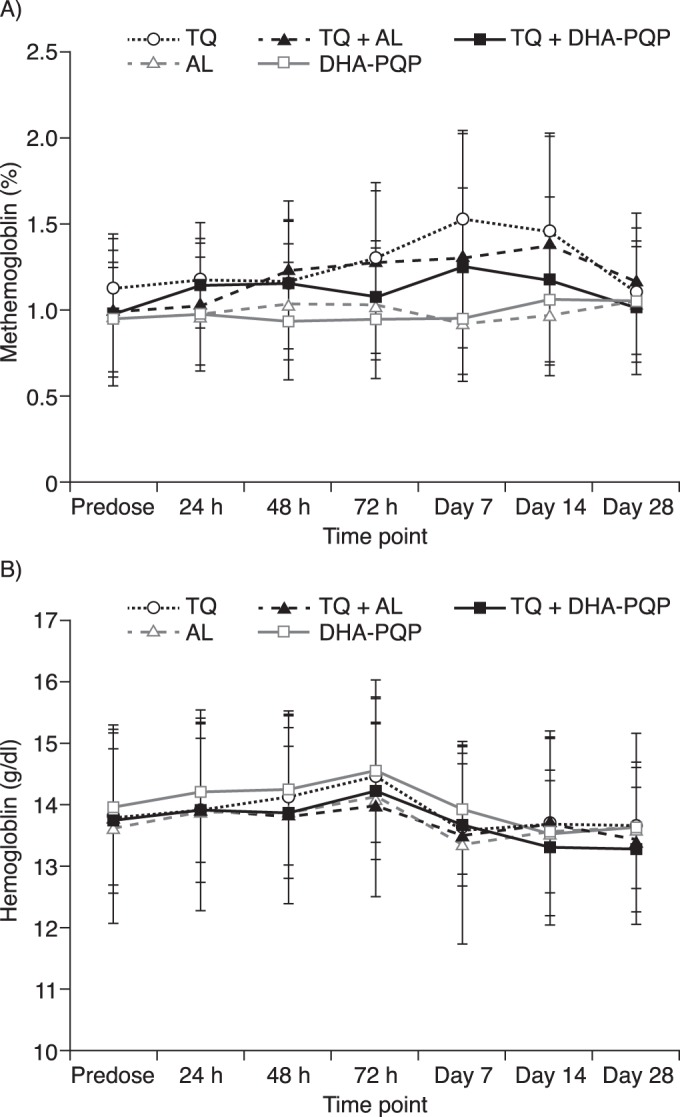
Mean methemoglobin levels as a percentage of hemoglobin (± standard deviations) (A) and mean hemoglobin levels (± standard deviations) (B) following dosing with tafenoquine (TQ), artemether-lumefantrine (AL), dihydroartemisinin-piperaquine (DHA-PQP), artemether-lumefantrine plus tafenoquine, or dihydroartemisinin-piperaquine plus tafenoquine.

Hematological laboratory evaluations showed no clinically relevant changes from the baseline for any cohort, except for methemoglobin levels, which were elevated at days 7 to 14 in the cohorts receiving tafenoquine (Fig. 2A). The maximum methemoglobin level was 3.2% with tafenoquine, 4.1% with tafenoquine plus artemether-lumefantrine, and 2.6% with tafenoquine plus dihydroartemisinin-piperaquine. Methemoglobin levels below 10% are of no clinical concern, and there were no clinical signs or symptoms associated with increased methemoglobin levels, which had returned to baseline levels by day 28. There was no additional effect of coadministration of dihydroartemisinin-piperaquine or artemether-lumefantrine with tafenoquine on methemoglobin levels (Fig. 2A). There was no decline in hemoglobin levels of >2.0 g/dl in any cohort and no additional effect of tafenoquine coadministration with ACTs on hemoglobin levels (Fig. 2B). Sequencing for G6PD genotyping was performed for all females. One subject was found to carry the A-deficient heterozygous genotype (376A>G; 202G>A), although the subject met the inclusion criteria for G6PD enzyme activity. Her baseline hemoglobin level was 12.8 g/dl, with a maximum decline of 1.3 g/dl (nadir of 11.5 g/dl on day 14). She did not report any symptoms of hemolytic anemia.

### Pharmacodynamic evaluation.

**FIG 3:**
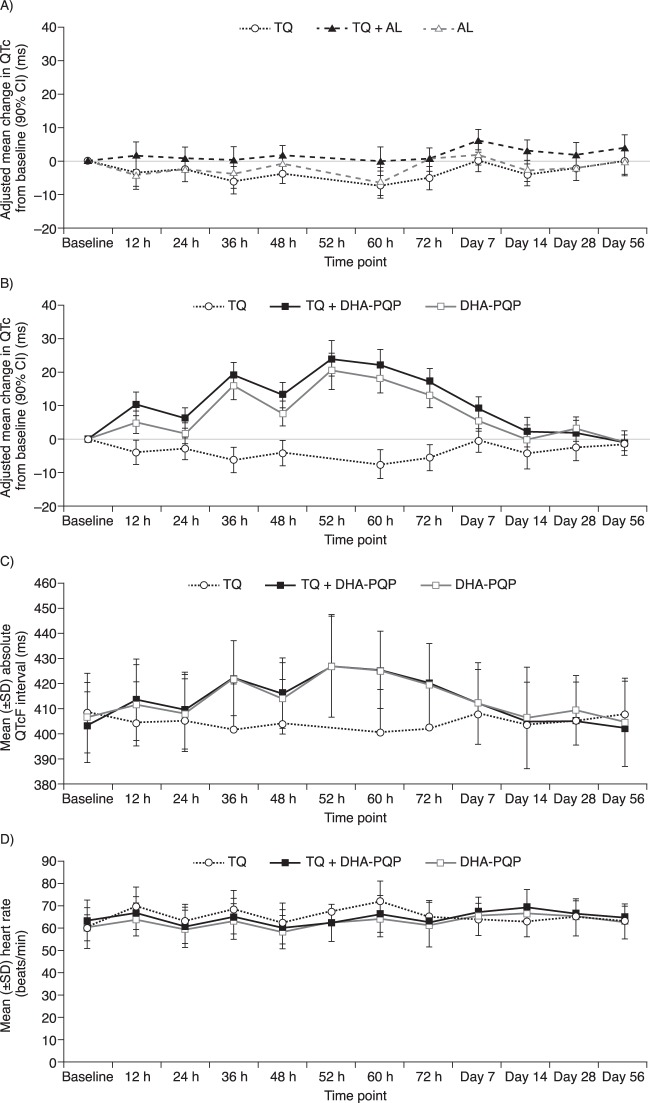
(A) Adjusted mean change (90% CI) in QTcF from baseline following administration of artemether-lumefantrine (AL), artemether-lumefantrine plus tafenoquine (TQ), or tafenoquine alone. (B) Adjusted mean change (90% CI) in QTcF from baseline following administration of dihydroartemisinin-piperaquine (DHA-PQP), dihydroartemisinin-piperaquine plus tafenoquine, or tafenoquine alone. (C) Mean absolute QTcF (and standard deviations) following administration of dihydroartemisinin-piperaquine, dihydroartemisinin-piperaquine plus tafenoquine, or tafenoquine alone. (D) Mean heart rate following administration of dihydroartemisinin-piperaquine, dihydroartemisinin-piperaquine plus tafenoquine, or tafenoquine alone.

There was no effect of tafenoquine alone on QTcF (Fig. 3A and B), and no subject in the tafenoquine-only group had a maximum QTcF interval of >450 ms or a maximum change in the QTcF interval from the baseline of >30 ms. Artemether-lumefantrine with or without tafenoquine had no effect on QTcF versus the baseline (Fig. 3A).

The mean QTcF was increased from the baseline following dihydroartemisinin-piperaquine treatment with or without tafenoquine (Fig. 3B). The maximum mean increase in QTcF from the baseline was ∼20 ms at 52 h post-first dose (i.e., around the *C*_max_ of the third dose of DHA-PQP) (Fig. 3B). However, no subject in any treatment group had a maximum QTcF interval of >480 ms, and none had a maximum change in the QTcF interval from the baseline of >60 ms. Overall, the absolute QTcF and the mean change in the QTcF versus the baseline were similar for dihydroartemisinin-piperaquine versus tafenoquine plus dihydroartemisinin-piperaquine (Fig. 3B and C). Analysis of treatment difference by time found an additional prolongation of QTcF from the baseline with tafenoquine coadministration versus dihydroartemisinin-piperaquine alone at 12 h (5.63 ms; 90% CI, 0.76 to 10.51 ms), 24 h (4.55 ms; 90% CI, 0.22 to 8.89 ms), and 48 h (5.63 ms; 90% CI, 0.38 to 10.89 ms) post-first dose (see Table SA2 in the supplemental material). However, the overall observed differences were small, and confidence intervals of the treatment groups overlapped. At 52 h post-first dose (i.e., around the *C*_max_ of the third dose of DHA-PQP), the absolute difference was 3.8 ms (95% CI, −3.6 to 11.2 ms). The observed differences in QTcF from the baseline following dihydroartemisinin-piperaquine treatment with or without tafenoquine were not explained by mean changes in heart rate over time (Fig. 3D).

### Pharmacokinetics.

**FIG 4:**
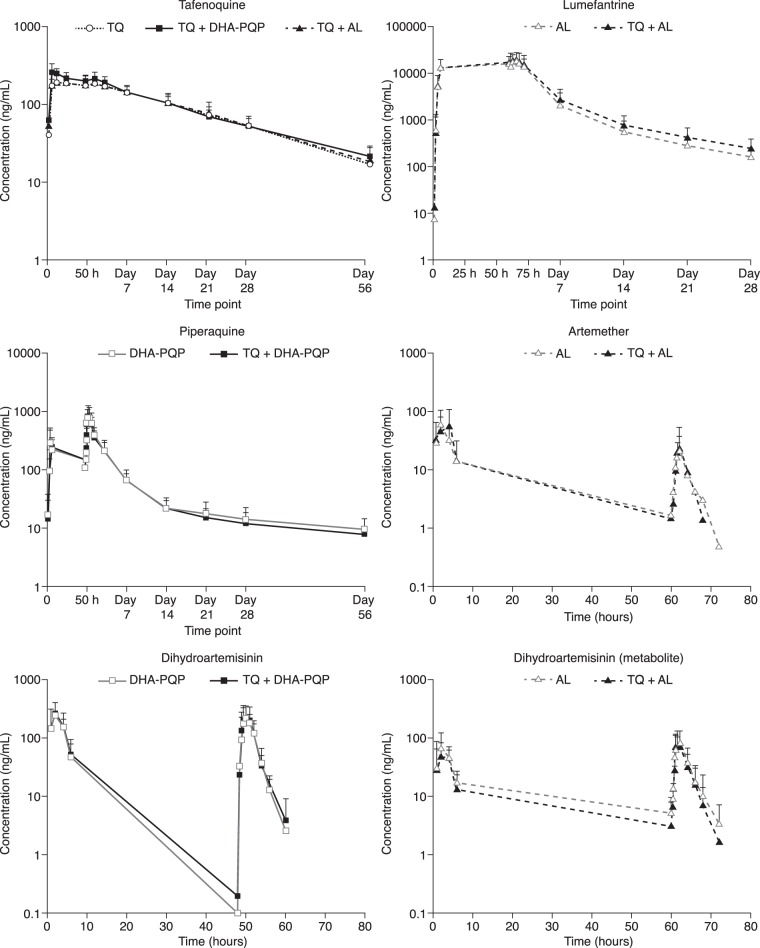
Concentration-time profiles for tafenoquine (TQ), lumefantrine, piperaquine, artesunate, and dihydroartemisinin (directly dosed or as a metabolite as artemether) dosed as tafenoquine plus dihydroartemisinin-piperaquine (DHA-PQP); tafenoquine plus artemether-lumefantrine (AL); or tafenoquine, dihydroartemisinin-piperaquine, or artemether-lumefantrine alone.

At least partial PK concentration profiles were available for all subjects and were included in the concentration profile plots (Fig. 4). The concentration-time profiles were broadly similar for ACTs with or without tafenoquine and for tafenoquine with or without ACT. Pharmacokinetic parameters were derived for all but three subjects (two who received artemether-lumefantrine and one who received dihydroartemisinin-piperaquine). For the primary analysis (Tables 3 to 5), a further three subjects were excluded: one subject receiving artemether-lumefantrine, because of duplicate sampling times, and two receiving artemether-lumefantrine plus tafenoquine, because of incomplete dosing.

**TABLE 3:** Pharmacokinetic parameters for and analysis of tafenoquine following administration of tafenoquine with or without dihydroartemisinin-piperaquine or artemether-lumefantrine^*a*^

Analyte and treatment group or parameter	Geometric mean AUC_0–∞_ (ng · h/ml)	Geometric mean AUC_0–last_ (ng · h/ml)	Geometric mean *C*_max_ (ng/ml)	Geometric mean *t*_1/2_ (h)	Median *T*_max_ (h) (range)
Tafenoquine					
TQ + DHA-PQP (*n* = 24)	109,333.7	93,809.3	274.7	483.9	6.0 (6.0–23.0)
TQ (*n* = 24)	97,195.5	88,283.9	199.6	375.2	12.1 (6.0–72.0)
CVb (%)	23.4	21.6	20.7	16.3	
GMR (90% CI)	1.12 (1.01, 1.26)	1.06 (0.96, 1.18)	1.38 (1.25, 1.52)	1.29 (1.19, 1.40)	
Tafenoquine					
TQ + AL (*n* = 22)	102,328.4	91,119.3	208.4	396.5	12.1 (2.0–60.0)
TQ (*n* = 24)	97,195.5	88,283.9	199.6	375.2	12.1 (6.0–72.0)
CVb (%)	26.2	24.1	19.9	15.5	
GMR (90% CI)	1.05 (0.93, 1.20)	1.03 (0.92, 1.16)	1.04 (0.95, 1.15)	1.06 (0.98, 1.14)	

a CVb, between-subject variability; GMR, geometric mean ratio.

**TABLE 4:** Pharmacokinetic parameters and analysis for dihydroartemisinin and piperaquine following administration of dihydroartemisinin-piperaquine with or without tafenoquine^*a*^

Analyte and treatment group or parameter	Geometric mean AUC_0–τ_ (ng · h/ml)	Geometric mean AUC_0–last_ (ng · h/ml)	Geometric mean *C*_max_ (ng/ml)	Geometric mean *t*_1/2_ (h)	Median *T*_max_ (h) (range)
Dihydroartemisinin					
TQ + DHA-PQP (*n* = 24)	761.2	748.9	262.5	1.7	2.0 (1.0–6.0)
DHA-PQP (*n* = 23)	758.0	749.5	277.5	1.6	2.0 (1.0–6.0)
CVb (%)	44.0	43.9	50.9	36.7	
Ratio (90% CI)	1.00 (0.82, 1.24)	1.00 (0.81, 1.23)	0.95 (0.75, 1.20)	1.05 (0.88, 1.25)	
Piperaquine					
TQ + DHA-PQP (*n* = 24)	9,206.5	35,660.1	840.5	360.4	4.0 (3.0–8.0)
DHA-PQP (*n* = 23)	9,815.6	37,358.0	928.2	382.2	4.0 (3.0–8.0)
CVb (%)	30.2	32.5	36.8	89.5	
Ratio (90% CI)	0.94 (0.81, 1.08)	0.95 (0.82, 1.11)	0.91 (0.76, 1.08)	0.94 (0.65, 1.37)	

a CVb, between-subject variability.

**TABLE 5:** Pharmacokinetic parameters and analysis for artemether, dihydroartemisinin (metabolite), and lumefantrine following administration of artemether-lumefantrine with or without tafenoquine^*d*^

Analyte and treatment group or parameter	Geometric mean AUC_0–τ_ (ng · h/ml)	Geometric mean AUC_0–last_ (ng · h/ml)	Geometric mean *C*_max_ (ng/ml)	Geometric mean *t*_1/2_ (h)	Median *T*_max_ (h) (range)
Artemether					
TQ + AL (*n* = 22)	186.2^*a*^	38.8	23.2	1.5^*a*^	2.0 (1.0–6.0)^*c*^
AL (*n* = 21)	103.0^*b*^	37.7	22.4	2.0^*b*^	2.0 (1.0–8.0)
CVb (%)	62.8	216.2	80.9	76.3	
Ratio (90% CI)	1.81 (1.06, 3.10)	1.03 (0.52, 2.04)	1.03 (0.71, 1.49)	0.76 (0.41, 1.44)	
Dihydroartemisinin (metabolite)					
TQ + AL (*n* = 22)	226.8	239.3	86.8	1.8^*c*^	1.9 (2.0–6.0)
AL (*n* = 21)	294.6	293.3	103.3	2.2	2.0 (1.0–8.0)
CVb (%)	45.0	46.8	53.5	32.9	
Ratio (90% CI)	0.77 (0.62, 0.96)	0.82 (0.65, 1.02)	0.84 (0.65, 1.09)	0.85 (0.72, 1.00)	
Lumefantrine					
TQ + AL (*n* = 22)	196,498.6	1,043,185.4	20,445.0	197.9	5.9 (0–12.0)
AL (*n* = 21)	174,602.3	808,244.6	18,911.0	164.7	4.0 (0–12.0)
CVb (%)	52.3	61.4	47.2	30.0	
Ratio (90% CI)	1.13 (0.87, 1.45)	1.29 (0.97, 1.73)	1.08 (0.86, 1.36)	1.20 (1.03, 1.40)	

a *n* = 5.

b *n* = 12.

c *n* = 21.

d CVb, between-subject variability.

### Tafenoquine pharmacokinetics.

Coadministration of dihydroartemisinin-piperaquine caused modest increases in tafenoquine *C*_max_ of 38% (90% CI, 25 to 52%), AUC_0–∞_ of 12% (90% CI, 1 to 26%), and *t*_1/2_ of 29% (90% CI, 19 to 40%), and the *T*_max_ was approximately halved (6.0 versus 12.1 h), with no impact on the AUC_0–last_ (Table 3). There was no effect of artemether-lumefantrine on the pharmacokinetics of tafenoquine, as can be seen for exposure comparisons across all parameters (Table 3).

### Dihydroartemisinin-piperaquine pharmacokinetics.

There was no effect of tafenoquine coadministration on the plasma pharmacokinetics of dihydroartemisinin (Table 4). High between-subject variability (50.9%) (Table 4) resulted in wide 90% confidence intervals around the dihydroartemisinin *C*_max_. There was no effect of tafenoquine coadministration on the plasma pharmacokinetics of piperaquine (Table 4).

### Artemether-lumefantrine pharmacokinetics.

The lumefantrine *t*_1/2_ was increased by 20% (90% CI, 3 to 40%) with tafenoquine coadministration (Table 5). Although the confidence intervals were wide because of the large interpatient variation, the point estimates indicate that there was no clinically relevant effect of tafenoquine coadministration on the lumefantrine AUC_0–τ_, AUC_0–last_, or *C*_max_ (Table 5).

Tafenoquine coadministration increased the artemether AUC_0–τ_ by 81% (90% CI, 6 to 210%) and decreased the dihydroartemisinin (metabolite) AUC_0–τ_ by 23% (90% CI, 4 to 38%) (Table 5). However, the AUC_0–τ_ was calculated for only 12/22 subjects receiving artemether-lumefantrine and 5/24 subjects receiving tafenoquine plus artemether-lumefantrine. The extremely short *t*_1/2_ for artemether makes it difficult to extrapolate AUC_0–τ_ values, and so in this case, the AUC_0–last_ is a more relevant comparison for determining the effect of tafenoquine on artemether pharmacokinetics. There was no effect of tafenoquine coadministration on the AUC_0–last_, *C*_max_, or *t*_1/2_ of artemether (Table 5). There was no effect of tafenoquine coadministration on dihydroartemisinin (metabolite) exposure (Table 5).

## DISCUSSION

Clinical trials of tafenoquine in combination with ACTs for the prevention of P. vivax relapse are needed in regions where chloroquine no longer retains antimalarial efficacy against this parasite or in countries where chloroquine is (or will be) no longer the first-line therapy for the treatment of P. vivax malaria. This study investigated potential drug interactions between tafenoquine at a clinical dose of 300 mg and two ACTs, dihydroartemisinin-piperaquine and artemether-lumefantrine.

The greatest potential for drug-drug interactions with antimalarial drugs is via the cytochrome P450 enzyme family, and this pathway is involved in the metabolism of all the antimalarial drugs tested in this study. Piperaquine is a cytochrome P450 3A4 (CYP3A4) inhibitor and a substrate of CYP34A (14). Piperaquine plasma concentrations may be increased when it is coadministered with CYP3A4 inhibitors, with a potential exacerbation of the effect on QTc prolongation. Significant decreases in artemether, dihydroartemisinin (metabolite), and lumefantrine concentrations are observed with the coadministration of CYP3A4 inducers (15). However, based on all the available *in vitro* and *in vivo* data, tafenoquine is unlikely to perpetrate any drug-drug interaction by CYP inhibition (CYP3A4, CYP1A2, CYP2C9, and also CYP2D6) or as a result of the induction of CYP3A4 (tafenoquine investigator's brochure, document reference GM2007/00152/09 version 13, 7 December 2015; GlaxoSmithKline). This clinical study confirms the predicted lack of clinically significant drug interactions of tafenoquine coadministration with dihydroartemisinin-piperaquine or artemether-lumefantrine.

The study enrolled an adequate number of subjects based on between-subject coefficients of variation in the pharmacokinetic parameters for the analytes. For the analytes with high variability, the confidence intervals may be very wide and thus include 1.0, even though the point estimates indicate modest changes. For example, a 29% increase in the lumefantrine AUC_0–last_ was seen when artemether-lumefantrine was coadministered with tafenoquine. There is high variability in the bioavailability and, thus, the systemic concentrations of lumefantrine, as reported in literature (15, 16). Greater lumefantrine exposures than those obtained in the present study were reported previously, for example, a mean *C*_max_ of 25,700 ng/ml (17), compared to a geometric mean *C*_max_ of 20,445 ng/ml in the present study. With the short treatment duration, these minor increases in exposure are therefore not thought to be clinically relevant.

The frequency of adverse events with tafenoquine alone in this study was low (12.5%; 3/24). The frequency of headache was increased when tafenoquine was coadministered with artemether-lumefantrine, and headache was the most common adverse event with tafenoquine in this study across all cohorts (11.1%; 8/72). Headache was also the most common adverse event with 300 mg tafenoquine in a phase IIb clinical trial (18%; 10/57) (4). Otherwise, tafenoquine coadministration with ACT compared to administration of ACT alone did not result in an increase in the number of adverse events and did not affect the nature of the adverse events experienced. There were no serious adverse events experienced by any subject across the three cohorts that received tafenoquine.

Elevations in methemoglobin levels are known to occur with tafenoquine, and mild increases in methemoglobin levels (maximum, 4.1%) were observed in this study. However, methemoglobin levels were not increased with the coadministration of tafenoquine plus ACT. In a study of tafenoquine with or without chloroquine in healthy volunteers, all three subjects who received the combination had methemoglobin values of >10% (11.0 to 13.2%), with no clinical symptoms (11). However, in a phase IIb dose-ranging clinical trial, there were no cases of methemoglobinemia with tafenoquine at doses of up to 600 mg in combination with chloroquine (4). Thus, it appears that the elevations of methemoglobin levels are not a clinical concern for the coadministration of tafenoquine with chloroquine or the ACTs tested in this study. Notably, there was no decline in hemoglobin levels of >2 g/dl in any cohort.

Tafenoquine alone had no effect on QTcF prolongation in this study, and these findings are consistent with data from preclinical studies and a formal thorough QT study and with clinical data for P. vivax patients, which show that tafenoquine has a low potential for QTcF prolongation (4, 18). A previous drug interaction study demonstrated no additional effect of tafenoquine on QTcF when tafenoquine was coadministered with chloroquine (11), and this has been supported by clinical evidence from a large phase IIb study including tafenoquine doses of up to 600 mg (4). In the present study, there was no clinically relevant effect of artemether-lumefantrine with or without tafenoquine coadministration on QTcF.

Dihydroartemisinin-piperaquine is known to cause a prolongation of the QT interval (19), although there have been no reported cases of Torsades de Pointes. In this study, one subject receiving dihydroartemisinin-piperaquine experienced cardiac arrest, possibly caused by high vagal tone, nausea, and diarrhea. The principal investigator and local cardiologist felt that dihydroartemisinin-piperaquine could also have contributed to this event. QTcF prolongation was observed for subjects receiving dihydroartemisinin-piperaquine in this study, although changes in QTcF were not clinically significant. The pharmacokinetics observed for piperaquine in this study were similar to those previously reported for multiple dosing in healthy volunteers (20). Piperaquine exposure is known to be increased with the consumption of a high-fat, high-calorie meal (21). However, the low-fat food provided in the present study did not appear to have any noticeable effect on piperaquine pharmacokinetics compared to data from previous studies of unfed healthy volunteers (20). In the present study, no subject receiving dihydroartemisinin-piperaquine had a QTcF prolongation of >480 ms or had a difference from the baseline of >60 ms, and this is consistent with data from previous studies of unfed healthy volunteers (22). With tafenoquine plus dihydroartemisinin-piperaquine, there were small increases in the QTcF (4.55 to 5.63 ms) at 12, 24, and 48 h post-first dose versus dihydroartemisinin-piperaquine alone but no difference at 52 h post-first dose, which is when any effect might have been expected given the pharmacodynamics of dihydroartemisinin-piperaquine. Thus, coadministration of tafenoquine and dihydroartemisinin-piperaquine had no additional clinically important effect on QTcF prolongation, and no dose adjustment is necessary. There are no reported data for QTc prolongation following dihydroartemisinin-piperaquine treatment in patients with P. vivax malaria. However, for Plasmodium falciparum malaria, QTc prolongation is more severe and more frequent in patients than in healthy volunteers, possibly because of increased piperaquine exposure in malaria patients (22). The potential for QTc prolongation will therefore be closely monitored in planned larger studies of tafenoquine plus dihydroartemisinin-piperaquine in malaria patients in Asia.

The observed pharmacokinetics for tafenoquine administered alone in this study were similar to those reported previously in studies of volunteers (11, 18). Coadministration of dihydroartemisinin-piperaquine increased tafenoquine exposure modestly, with the *C*_max_ being increased by 38%. However, the tafenoquine geometric mean *C*_max_ (274.7 ng/ml) observed following dihydroartemisinin-piperaquine coadministration in this study was still well below that observed for treatment with 600 mg tafenoquine (422 ng/ml) (18) in P. vivax malaria patients (4), so this increased exposure to tafenoquine is of no clinical concern in individuals with normal G6PD levels. In G6PD-deficient individuals, the risk of hemolysis appears to increase with the tafenoquine dose (2), although such patients should be excluded from tafenoquine treatment.

In a previous study, coadministration of chloroquine (a 4-aminoquinoline, like piperaquine) also increased the tafenoquine *C*_max_ by 38% versus tafenoquine alone (11). Studies with primaquine (an 8-aminoquinline, like tafenoquine) also showed increases in the primaquine *C*_max_ with coadministration with chloroquine (63% increase) (23) or dihydroartemisinin-piperaquine (48% increase) (24). Thus, the coadministration of 8-aminoquinolines (tafenoquine and primaquine) with 4-aminoquinolines (chloroquine and piperaquine) appears to produce a consistent increase in exposure for the 8-aminoquinoline without a reciprocal effect on the pharmacokinetics of the 4-aminoquinoline. One explanation could be competition between the two molecular classes for monoamine oxidase or cytochrome P450-mediated metabolism, with elimination of the 8-aminoquinolines being decreased, thus boosting exposure. However, this does not explain the effect of piperaquine on the increase in the tafenoquine *C*_max_, given the very slow elimination of tafenoquine. Notably, exposure to the primaquine metabolite carboxyprimaquine is also enhanced by 21% in combination with chloroquine and by 33% with dihydroartemisinin-piperaquine, reflecting the multiple metabolic pathways and metabolites associated with primaquine (23, 24). There are no major metabolites of tafenoquine.

Tafenoquine coadministration increased the artemether AUC_0–τ_ by 81%. However, in subjects receiving artemether-lumefantrine alone or with tafenoquine, the exposure parameter AUC_0–τ_ was calculated for only a limited number of subjects after the last dose of artemether-lumefantrine. This was clearly attributable to the short *t*_1/2_ of artemether (∼2 h). Thus, the exposure comparisons were based primarily on AUC_0–last_ values. The *C*_max_ and AUC_0–last_ estimates for artemether were in complete agreement with historically reported values (25–27). This lends further support for the use of AUC_0–last_ to compare exposures upon coadministration. There was no impact of tafenoquine coadministration on the artemether AUC_0–last_. Thus, no dose adjustment is deemed necessary for artemether-lumefantrine coadministered with tafenoquine. There was no effect of tafenoquine (8-aminoquinline) on lumefantrine (aryl-amino-alcohol) pharmacokinetics, and this is consistent with the lack of an effect of primaquine (8-aminoquinoline) on mefloquine (aryl-amino-alcohol) pharmacokinetics (28, 29).

Tafenoquine is being investigated as a single-dose therapy for the prevention of P. vivax relapse and will need to be coadministered with a blood schizonticide drug, either chloroquine or an ACT, depending on parasite drug resistance. This study demonstrates that there were no clinically significant drug interactions between tafenoquine and the first-line ACTs dihydroartemisinin-piperaquine and artemether-lumefantrine. Thus, no dosing adjustment is deemed necessary for the coadministration of these drugs with tafenoquine. There was no evidence of any increased safety risk, and known safety issues with each of the drugs tested were not exacerbated by their coadministration. Further clinical studies will investigate the combination of tafenoquine plus dihydroartemisinin-piperaquine in regions with chloroquine-resistant P. vivax.

## Supplementary Material

Supplemental material
