# Supplementary material for: Pharmacokinetic Interactions between Tafenoquine and Dihydroartemisinin-Piperaquine or Artemether-Lumefantrine in Healthy Adult Subjects
Source: Antimicrob Agents Chemother. 2016 Nov 21;60(12):7321–32. doi: 10.1128/AAC.01588-16 (PMC5119013; doi:10.1128/AAC.01588-16)
Supplement: Supplemental material [file supp_60_12_7321__index.html]

Supplemental material 

# Pharmacokinetic Interactions between Tafenoquine and Dihydroartemisinin-Piperaquine or Artemether-Lumefantrine in Healthy Adult Subjects

## Supplemental material

**Files in this Data Supplement:**

- Supplemental file 1 -

  Tables SA1 and SA2

  PDF, 289K
