# Supplementary material for: Pharmacokinetic Interactions between Tafenoquine and Dihydroartemisinin-Piperaquine or Artemether-Lumefantrine in Healthy Adult Subjects
Source: Antimicrob Agents Chemother. 2016 Nov 21;60(12):7321–32. doi: 10.1128/AAC.01588-16 (PMC5119013; doi:10.1128/AAC.01588-16)
Supplement: Supplemental material [file AAC.01588-16_zac012165760so1.pdf]

Table A1: Pharmacokinetic sampling times and assays by cohort

| Sampling Times | | Cohort 1 (TQ + DHA-PQP) | | Cohort 2 (TQ + AL) | | Cohort 3 (DHA-PQP) | | Cohort 4 (AL) | | Cohort 5 (TQ) | |
|---|---|---|---|---|---|---|---|---|---|---|---|
| **Day** | **Hour** | **# 2mL blood samples** | **Assays** | **# 2mL blood samples** | **Assays** | **# 2mL blood samples** | **Assays** | **# 2mL blood samples** | **Assays** | **# 2mL blood samples** | **Assays** |
| | **Pre-dose** | 2 | A, B | 2 | A' C | 1 | B | 1 | C | 1 | A |
| | **1** | 1 | B | 1 | C | 1 | B | 1 | C | | |
| **1** | **2** | 2 | A, B | 2 | A, C | 1 | B | 1 | C | 1 | A |
| | **4** | 1 | B | 1 | C | 1 | B | 1 | C | | |
| | **6** | 2 | A, B | 2 | A, C | 1 | B | 1 | C | 1 | A |
| | **12** | 1 | A | 1 | A | | | | | 1 | A |
| **2** | **24** | 1 | A | 1 | A | | | | | 1 | A |
| **3** | **48** | 2 | A, B | 1 | A | 1 | B | | | 1 | A |
| | **48.5** | 1 | B | | | 1 | B | | | | |

| Sampling Times | | Cohort 1 (TQ + DHA-PQP) | | Cohort 2 (TQ + AL) | | Cohort 3 (DHA-PQP) | | Cohort 4 (AL) | | Cohort 5 (TQ) | |
|---|---|---|---|---|---|---|---|---|---|---|---|
| **Day** | **Hour** | **# 2mL blood samples** | **Assays** | **# 2mL blood samples** | **Assays** | **# 2mL blood samples** | **Assays** | **# 2mL blood samples** | **Assays** | **# 2mL blood samples** | **Assays** |
| | **49** | 1 | B | | | 1 | B | | | | |
| | **49.5** | 1 | B | | | 1 | B | | | | |
| | **50** | 1 | B | | | 1 | B | | | | |
| | **51** | 1 | B | | | 1 | B | | | | |
| | **52** | 1 | B | | | 1 | B | | | | |
| | **54** | 1 | B | | | 1 | B | | | | |
| | **56** | 1 | B | | | 1 | B | | | | |
| | **60** | 2 | A, B | 2 | A, C | 1 | B | 1 | C | 1 | A |
| | **60.5** | | | 1 | C | | | 1 | C | | |
| | **61** | | | 1 | C | | | 1 | C | | |
| | **61.5** | | | 1 | C | | | 1 | C | | |

| Sampling Times | | Cohort 1 (TQ + DHA-PQP) | | Cohort 2 (TQ + AL) | | Cohort 3 (DHA-PQP) | | Cohort 4 (AL) | | Cohort 5 (TQ) | |
|---|---|---|---|---|---|---|---|---|---|---|---|
| **Day** | **Hour** | **# 2mL blood samples** | **Assays** | **# 2mL blood samples** | **Assays** | **# 2mL blood samples** | **Assays** | **# 2mL blood samples** | **Assays** | **# 2mL blood samples** | **Assays** |
| | **62** | | | 1 | C | | | 1 | C | | |
| | **64** | | | 1 | C | | | 1 | C | | |
| **4** | **66** | | | 1 | C | | | 1 | C | | |
| | **68** | | | 1 | C | | | 1 | C | | |
| | **72** | 2 | A, B | 2 | A, C | 1 | B | 1 | C | 1 | A |
| **7** | **-** | 2 | A, B | 2 | A, C | 1 | B | 1 | C | 1 | A |
| **14** | **-** | 2 | A, B | 2 | A, C | 1 | B | 1 | C | 1 | A |
| **21** | **-** | 2 | A, B | 2 | A, C | 1 | B | 1 | C | 1 | A |
| **28** | **-** | 2 | A, B | 2 | A, C | 1 | B | 1 | C | 1 | A |
| **56** | **-** | 2 | A, B | 1 | A | 1 | B | - | - | 1 | A |
| **∑ # samples** | | 34 | | 31 | | 21 | | 18 | | 13 | |

| Sampling Times | | Cohort 1 (TQ + DHA-PQP) | | Cohort 2 (TQ + AL) | | Cohort 3 (DHA-PQP) | | Cohort 4 (AL) | | Cohort 5 (TQ) | |
|---|---|---|---|---|---|---|---|---|---|---|---|
| **Day** | **Hour** | **# 2mL blood samples** | **Assays** | **# 2mL blood samples** | **Assays** | **# 2mL blood samples** | **Assays** | **# 2mL blood samples** | **Assays** | **# 2mL blood samples** | **Assays** |
| **∑ # draws** | | 23 | | 22 | | 21 | | 18 | | 13 | |

Plasma assays:

A = tafenoquine (TQ)

B = piperaquine (PQP), dihydroartemisinin (DHA)

C = artemether (A), dihydroartemisinin (DHA), lumefantrine (L).

T

Table A2: Change from baseline QTcF treatment difference for the interaction of tafenoquine with dihydroartemisinin-piperaquine

| Time point | Difference for (tafenoquine + dihydroartemisinin-piperaquine) – dihydroartemisinin-piperaquine (90% CI) |
|---|---|
| 12 h | 5.63 (0.76, 10.51) |
| 24 h | 4.55 (0.22, 8.89) |
| 36 h | 3.22 (–2.21, 8.64) |
| 48 h | 5.63 (0.38, 10.89) |
| 52 h | 3.80 (–3.59, 11.20) |
| 60 h | 4.06 (–1.95, 10.07) |
| 72 h | 4.01 (–1.18, 9.20) |
| Day 7 | 3.38 (–1.45, 8.22) |
| Day 14 | 2.30 (–3.84, 8.44) |
| Day 28 | –1.14 (–6.40, 4.13) |
| Day 56 | 1.15 (–2.99, 5.29) |
